# Severe lymphomatoid papulosis with lymphadenopathy, facial involvement, and a pathogenic STAT3 variant

**DOI:** 10.1016/j.jdcr.2026.04.023

**Published:** 2026-04-21

**Authors:** Hayden Christensen, Karamatullah Danyal, Melanie R. Bui

**Affiliations:** a Robert Larner MD College of Medicine, University of Vermont, Burlington, Vermont; b Department of Pathology, University of Vermont Medical Center, Burlington, Vermont; c Department of Dermatology, University of Vermont Medical Center, Burlington, Vermont

**Keywords:** CD30+ lymphoproliferative disorders, lymphomatoid papulosis, primary cutaneous anaplastic large cell lymphoma

## Introduction

Primary cutaneous CD30+ T-cell lymphoproliferative disorders (LPDs) are rare cutaneous T-cell lymphomas, encompassing a spectrum including lymphomatoid papulosis (LyP) and primary cutaneous anaplastic large-cell lymphoma (pc-ALCL).^1^ Although CD30+ LPDs carry an excellent prognosis, their clinical presentations vary.^2^

LyP is characterized by recurrent papulonodules commonly with scaling and ulceration.^3^ Lesions favor the trunk and extremities with rare acral, mucosal, or facial involvement.^4^

In contrast, pc-ALCL typically manifests as rapidly growing solitary or grouped nodules which frequently ulcerate.^3^^,^^5^ Lesions favor the head, neck, and extremities. While the disease often remains localized, up to 20% present with disseminated lesions, most commonly involving regional lymph nodes.^3^^,^^6^ Limited skin involvement confers a favorable prognosis, with 5-y survival of 95%.^2^

Borderline cases between LyP and pc-ALCL include growing or nonhealing lesions lacking clinicopathologic distinction.^7^

Histologically, LyP and pc-ALCL are often indistinguishable, both showing sheets of atypical CD30+ T-lymphocytes amid background inflammation. LyP comprises 5 subtypes (A-E), with a proposed follicular variant (F type).^7^

Types A and C contain scattered large, atypical cells, distinguished by a robust mixed inflammatory infiltrate in Type A. Type B shows smaller atypical cells with cerebriform nuclei. Type D shows epidermotropic infiltration of CD8+ lymphocytes, and the infiltrate in Type E is angioinvasive. Type F exhibits follicular involvement with features of other subtypes.

In LyP, 10-y survival approaches 100%,^2^^,^^6^ but secondary malignancy, most commonly Mycosis fungoides, pc-ALCL, or Hodgkin lymphoma occurs in 15% to 50% of patients.^2^^,^^4^, ^5^, ^6^

In CD30+ LPDs, genetic variants may inform diagnosis and pathogenesis. In one cohort, activating JAK-STAT mutations were identified in 50% of examined CD30+ LPDs.^8^ STAT3 is recurrently mutated in systemic anaplastic large cell lymphoma (ALCL), suggesting an independent oncogenic role. The D661 V mutation causes constitutive activation of STAT3 and is exceptionally rare among patients with LyP. Importantly, these variants carry no established prognostic significance.^8^

Here, we present a case of severe LyP with a pathogenic STAT3 variant.

## Case presentation

**Fig 1:**
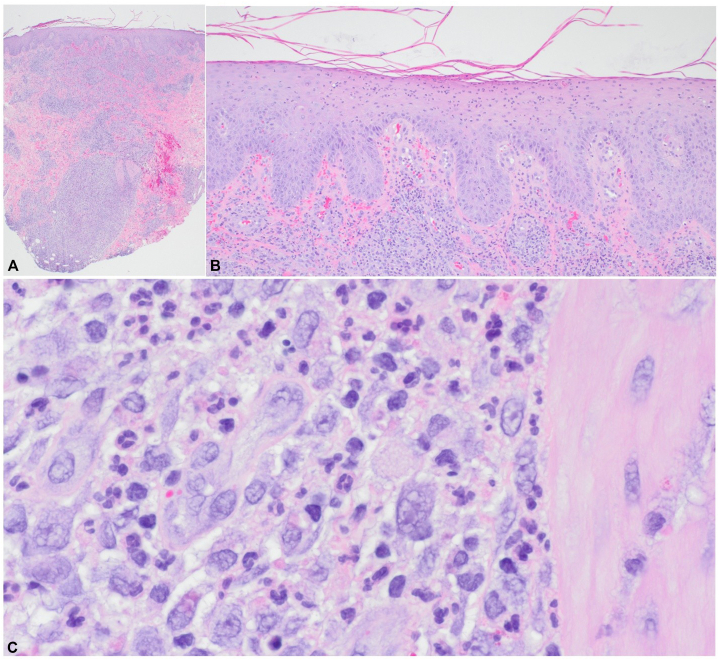
Histology of punch biopsy. **(A)** Hematoxylin and eosin low power (20x) view of the biopsy exhibits an inflammatory infiltrate involving the superficial papillary dermis and extending to the subcutis. **(B)** 40× magnification view exhibits a predominantly polymorphonuclear (neutrophilic) exocytosis into the epidermis with minimal interface or spongiotic change. Within the superficial papillary dermis, a Grenze Zone separates the dermal-epidermal junction from the mixed inflammatory infiltrate. **(C)** 600× magnification reveals the cytologic atypia of the lymphocytic component of the inflammatory infiltrate with vesicular nuclei, nuclear membrane irregularity, and increased nuclear to cytoplasmic ratio. When taken with Figure 2, this histology is most consistent with LyP type A. *LyP,* Lymphomatoid papulosis.

**Fig 2:**
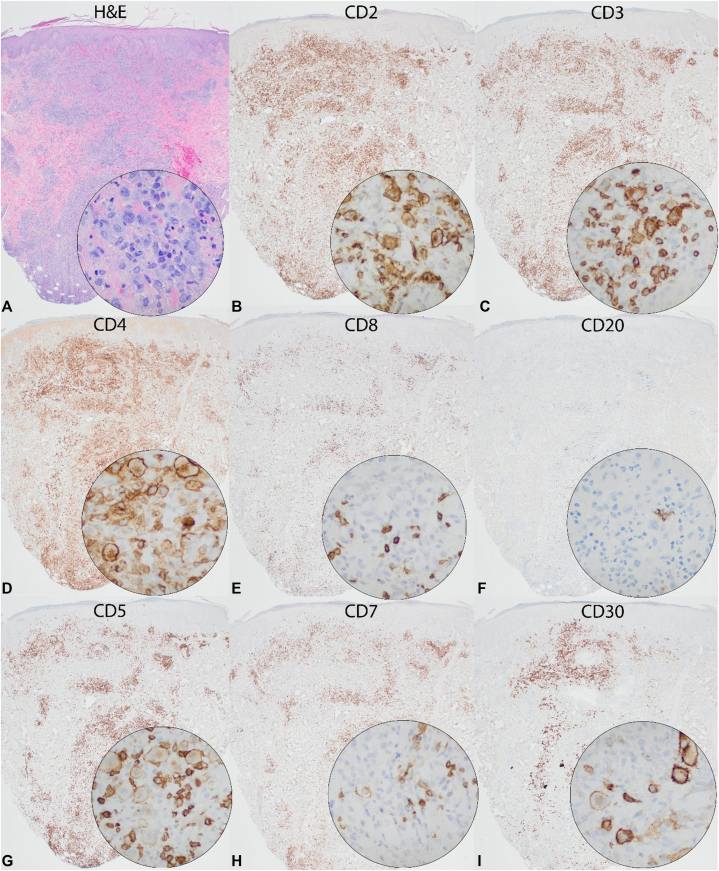
Immunohistochemical investigation of the punch biopsy specimen. **(A)** representative H&E section of biopsy. **(B** and **C)** CD2 and CD3 highlight the aberrant large T cell population. **(D** and **E)** CD4 and CD8 highlight T-helper lymphocytes and cytotoxic T cells respectively and shows the predominant population of aberrant cells to be off T helper phenotype. **(F)** shows rare B-lymphocytes. **(G)** exhibits diminished CD5 expression in T-lymphocytes. **(H)** CD7 is negative in the large, atypical lymphocytes. (I) exhibits CD30 positivity within the atypical slash aberrantly large T-lymphocytes.

A 49-year-old female with no significant medical history presented to primary care with 9 days of a progressive pruritic rash involving her trunk and extremities. A 10-day prednisone course yielded no improvement. A 4-mm punch biopsy of a lesion on her left anterior forearm demonstrated a dermal inflammatory infiltrate with enlarged, atypical CD30+ cells (Figs 1 and 2).

After biopsy, referral was placed to Hematology and Dermatology for evaluation. Further history revealed drenching night sweats and weight loss. However, she had initiated semaglutide within this period. Her cutaneous lesions continued spreading, but she noted some spontaneously resolved.

**Fig 3:**
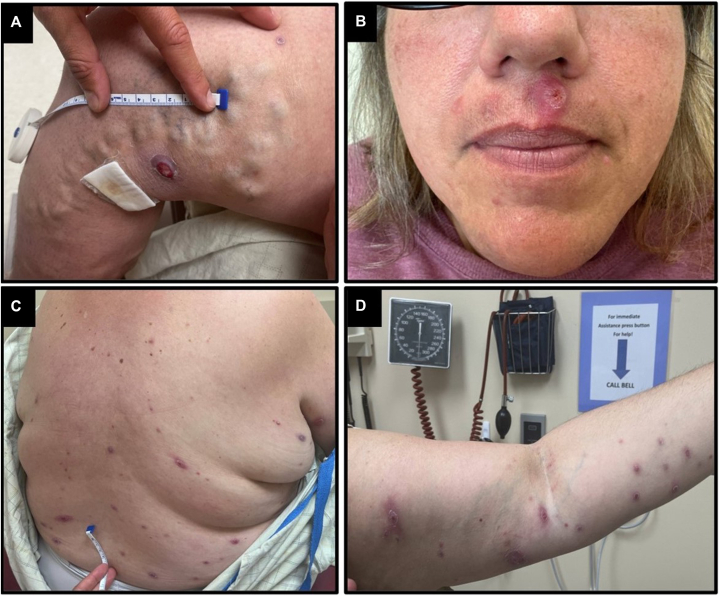
Initial disease involvement. Characteristic lesions at initial specialty referral. **(A)** 1.7 cm pink plaque on the right medial thigh with central ulceration. **(B)** Scaly pink papule of the upper cutaneous lip with surrounding erythema. **(C)** and **(D)** demonstrate scattered pink papules, plaques, and nodules, some with scale or central punctum of the back and left arm.

Examination showed extensive pink papules, plaques, and nodules scattered over the bilateral arms, axillae, back, groin, lower legs, and face with scale or ulceration (Fig 3).

A 2 cm, nontender left posterior cervical lymph node was palpated. Laboratory tests, including HIV, hepatitis B, and HTLV-1, were negative. Genetic testing of the skin biopsy demonstrated a STAT3 p.D661 V variant.

A whole-body positron emission tomography-computed tomography demonstrated multiple avid skin lesions consistent with an LPD, bilateral avid axillary and inguinal lymph nodes, and diffusely increased bone marrow activity. Bone marrow biopsy showed no evidence of an aberrant cell population. A limited left axillary lymph node biopsy showed small lymphocytes with clumped chromatin, and flow cytometry revealed no aberrant clones. Genetic testing of the lymph node detected no notable mutations.

Three months after initial presentation, the patient returned for follow-up at the completion of diagnostic testing. She reported regression of some lesions but continued to develop new ones, in clusters of 10 or more per week. Her night sweats resolved early in the disease course, and most original lesions were healing. Given the spontaneous regression of the lesions, negative laboratory, bone marrow, and lymph node findings, a diagnosis of LyP was favored over pc-ALCL. With diffuse cutaneous involvement and pruritis, a combination of low dose methotrexate and narrowband UVb (nb-UVb) therapy was initiated.

**Fig 4:**
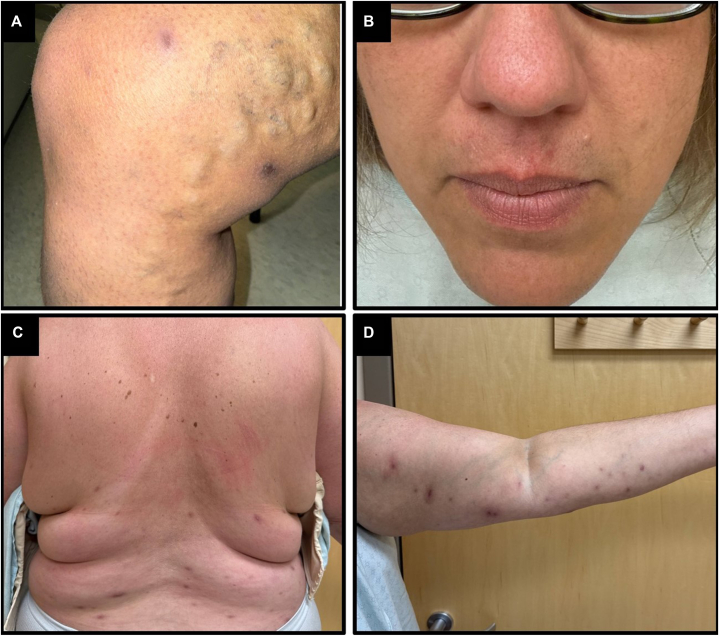
Skin involvement after 6 wks of treatment. Resolving papules, plaques, and ulcers of the right leg, and left arm **(A, D)**. Mild postinflammatory hyperpigmentation present on the back **(C)**, and a well-healed lip lesion **(B)**, with minimal residual disease.

Follow-up visit 6 weeks after therapy initiation demonstrated significant improvement of existing lesions (Fig 4), and limited development of new lesions.

No further lymphadenopathy was identified. The patient continues following with Dermatology and Hematology.

## Discussion

With an incidence estimated at 1.2 to 1.9 per million, LyP is a rare occurrence.^7^ Our case is further distinguished by the presence of a STAT3 D661 V mutation, an oncogenic mutation associated with large granular lymphocytic leukemia, but exceptionally rare in LyP.^8^ To our knowledge, only 2 prior LyP cases have reported STAT3 variants, neither of which carried the D661 V mutation.^8^ The identification of this oncogenic variant expands the genetic spectrum of the disease, highlights a potential therapeutic target, and underscores the need for further study into the clinical implications of STAT3 mutations in CD30+ LPDs.

In addition, the presence of nonimproving lesions, facial involvement, lymphadenopathy with night sweats and weight loss, raised concern for a malignant process. However, these “alarm” symptoms were confounded by our patient’s perimenopausal state and use of weight loss medication, respectively. Notably, these symptoms also resolved prior to treatment, lending further credence to a diagnosis of LyP over pc-ALCL. Though not definitive, certain risk factors for development of secondary malignancy have been described,^9^ with facial involvement a notably present risk factor in this patient.

Given semaglutide was initiated approximately 2 weeks prior to the onset of lesions, drug-induced CD30+ pseudolymphoma was considered. However, the clinical course was not consistent with this entity: several lesions self-resolved, new lesions developed for months after drug cessation, and there are no published reports linking glucagon-like peptide-1 receptor agonists to pseudolymphoma. Thus, this diagnosis was deemed unlikely.^10^

While Methotrexate and nb-UVb are effective in most patients at achieving complete or partial remission,^6^^,^^7^ these interventions have failed to demonstrate efficacy in preventing secondary cutaneous malignancies, or in altering the natural disease course, as regression is common upon termination of therapy.^2^^,^^7^

This case of severe LyP with alarm symptoms represents the challenging diagnostic overlap that CD30+ LPDs compose, highlighting the importance of robust clinical and historical examination to ensure diagnostic fidelity. Further, this case represents the importance of multidisciplinary observation for secondary malignancy development in LyP patients, especially when disease shows overlapping or uncommon features.

## Conflicts of interest

None disclosed.
